# Prognostic Role of Minimal Disseminated Disease and *NOTCH1/FBXW7* Mutational Status in Children with Lymphoblastic Lymphoma: The AIEOP Experience

**DOI:** 10.3390/diagnostics11091594

**Published:** 2021-09-01

**Authors:** Federica Lovisa, Ilaria Gallingani, Elena Varotto, Cristiano Pasin, Elisa Carraro, Barbara Michielotto, Anna Garbin, Carlotta Caterina Damanti, Marco Pizzi, Emanuele S. G. d’Amore, Matilde Piglione, Paola Muggeo, Salvatore Buffardi, Luciana Vinti, Veronica Maria Folsi, Daniela Onofrillo, Alessandra Biffi, Barbara Buldini, Marta Pillon, Lara Mussolin

**Affiliations:** 1 Maternal and Child Health Department, Padova University, 35128 Padova, Italy; federica.lovisa@unipd.it (F.L.); ilaria.gallingani@studenti.unipd.it (I.G.); cristiano.pasin.1@studenti.unipd.it (C.P.); barbara.michielotto@unipd.it (B.M.); anna.garbin.1@studenti.unipd.it (A.G.); carlottacaterina.damanti@studenti.unipd.it (C.C.D.); alessandra.biffi@unipd.it (A.B.); barbara.buldini@unipd.it (B.B.); 2 Istituto di Ricerca Pediatrica Città della Speranza, 35127 Padova, Italy; elena.varotto_01@aopd.veneto.it; 3 Pediatric Hematology, Oncology and Stem Cell Transplant Division, Padova University Hospital, 35128 Padova, Italy; elisa.carraro87@gmail.com (E.C.); marta.pillon@unipd.it (M.P.); 4 General Pathology and Cytopathology Unit, Department of Medicine-DMED, University of Padova, 35121 Padova, Italy; marco.pizzi.1@unipd.it; 5 Department of Pathology, San Bortolo Hospital, 36100 Vicenza, Italy; emanuele.damore@tin.it; 6 Paediatric Haematology, Department of Paediatrics, University Hospital Città della Salute e della Scienza di Torino, 10126 Torino, Italy; matilde.piglione@unito.it; 7 Pediatric Oncology and Hematology Unit, University Hospital of Policlinico, 70120 Bari, Italy; paola.muggeo@gmail.com; 8 Department of Paediatric Haemato-Oncology, Santobono-Pausilipon Children’s Hospital, 80123 Napoli, Italy; salvatorebuffardi@hotmail.it; 9 Department of Pediatric Hemato-Oncology, Ospedale Bambino Gesù, 00165 Rome, Italy; luciana.vinti@opbg.net; 10 Department of Pediatrics, Pediatrics Clinic, Spedali Civili of Brescia, 25123 Brescia, Italy; veronicafolsi@gmail.com; 11 Paediatric Haemato-Oncology Unit, Hematology Department, Hospital of Pescara, 65124 Pescara, Italy; danielaonofrillo@gmail.com

**Keywords:** lymphoblastic lymphoma, minimal disseminated disease, *NOTCH1/FBXW7*, child

## Abstract

*NOTCH1/FBXW7* (N/F) mutational status at diagnosis is employed for T-cell lymphoblastic lymphoma (T-LBL) patients’ stratification in the international protocol LBL 2018. Our aim was to validate the prognostic role of Minimal Disseminated Disease (MDD) alone and in combination with N/F mutational status in a large retrospective series of LBL pediatric patients. MDD was analyzed in 132 bone marrow and/or peripheral blood samples by flow cytometry. Mutations in N/F genes were analyzed on 58 T-LBL tumor biopsies. Using the previously established cut-off of 3%, the four-year progression-free survival (PFS) was 57% for stage I–III patients with MDD ≥ 3% versus 80% for patients with MDD inferior to cut-off (*p* = 0.068). We found a significant worsening in the four-year PFS for nonmutated (51 ± 12%) compared to mutated patients (100%, *p* = 0.0013). Combining MDD and N/F mutational status in a subgroup of available cases, we found a statistically significant difference in the four-year PFS for different risk groups (*p* = 0.0012). Overall, our results demonstrate that N/F mutational status has a more relevant prognostic value than MDD at diagnosis. However, the combination of N/F mutations with MDD analysis could identify patients with very aggressive disease, which might benefit from a more intensive treatment.

## 1. Introduction

Pediatric lymphoblastic lymphoma (LBL) represents the second most frequent non-Hodgkin lymphoma (NHL) subtype and accounts for approximately 25–35% of all NHL diagnosed in childhood and adolescence [1]. Most of LBLs are of T-lymphoblastic origin (70–80%), with 20–25% arising from B cell precursors (pB-LBL) [2,3].

During the last few years, great progress has been made by the scientific community in the study of pediatric LBL, and this allowed not only to improve our knowledge about the disease biology but also to design clinical and diagnostic strategies leading to event-free survival (EFS) and overall survival (OS) probabilities exceeding 80% [4,5]. Despite this, survival of patients with refractory or relapsed disease still remains poor [6]. In fact, valid prognostic stratification systems that allow effective treatment tailoring are currently missing, and this is basically due to the lack of clinical and laboratory prognostic factors. Recently, the study of minimal disseminated disease (MDD) and specifically the genetic analysis of the mutational status of *NOTCH1/FBXW7* (N/F) genes proved to be promising candidates for the prognostic evaluation of LBL patients [7,8,9,10].

The first data about the prognostic value of MDD in pediatric LBL emerged from the study by Coustan-Smith et al. in which the presence of submicroscopic disease in bone marrow (BM) and peripheral blood (PB), measured by flow cytometry, was associated with a poorer outcome [7]. Similar findings were later obtained also by our group, showing that a 3% cut-off on BM/PB involvement at diagnosis was able to identify patients in disease stages I–III characterized by a significantly worse prognosis [8].

Concerning N/F mutational status, two large independent studies reported that patients with T-LBL carrying mutations of NOTCH1 pathway are characterized by a more favorable prognosis than wild-type patients and that N/F mutational status represents an independent prognostic factor in multivariate analysis [9,10].

However, the combined significance of MDD and N/F mutations has never been investigated so far in T-LBL, mostly due to fact that this analysis relies on the availability of both the tumor tissue and BM and/or PB samples at diagnosis.

In light of this, the present study aims at defining the prognostic significance of MDD, assessed by multiparametric flow cytometry (MFC), in combination with N/F mutational status in the Italian cohort of pediatric T-LBL patients treated in clinical centers affiliated to the Associazione Italiana di Ematologia e Oncologia Pediatrica (AIEOP). The final goal of this study is to identify biological risk groups of patients which might be offered for alternative treatment approaches, both to reduce long-term toxicity and to effectively cure those at higher risk of treatment failure.

## 2. Materials and Methods

### 2.1. Patients, Samples and Treatment Protocol

This retrospective study included a cohort of 132 LBL pediatric patients (107 T-LBL and 25 pB-LBL) enrolled between June 2000 and January 2020, for which BM and/or PB at diagnosis were available to perform MDD analysis. For 58/107 T-LBL cases, the tumor tissue was also available for N/F mutational analysis. All patients were treated according to ALL-BFM-like therapeutic strategies. In particular, 103 patients were treated in the international EURO-LB02 protocol [5], whereas 29 in the AIEOP LNH-97 protocol [11]. The study was approved by the ethics committees or by the internal review committees of each participating institution and the informed consent of the parents or legal guardians was obtained before patients’ enrollment.

The diagnosis of LBL was established from clinical, histological, and immunohistochemistry findings. Tumors were classified as pB- or T-lineage LBL according to WHO guidelines [12]. In all cases, the histological diagnosis was centrally reviewed.

### 2.2. Multiparametric Flow Citometry Analysis of MDD

MDD analysis was conducted at the Pediatric Hemato-Oncology laboratory of the Department of Women’s and Children’s Health (University of Padua). The laboratory is the national reference center for molecular diagnostics of pediatric lymphomas enrolled in the AIEOP treatment protocols.

PB and BM samples were processed and analyzed as previously described [8,13,14]. Briefly, we performed immunophenotypic studies at diagnosis on erythrocyte-lysed whole BM/PB samples delivered from AIEOP centers at ambient temperature within 24–48 h of collection. We incubated 700,000 nucleated cells per analysis at room temperature for 10 min in the dark with different 6- or 8-color combinations (FITC/PE/PE-CY5/PE-CY7/APC/APC-CY7 or FITC/PE/PE-CY5/PE-CY7/APC/APC-CY7/V450/V500) of directly conjugated monoclonal antibodies (MoAbs), including T- and B-lineage markers. Samples were then lysed using 3 mL of NH_4_Cl (Carlo Erba Reagents, Cornaredo, Italy), washed in phosphate-buffered saline (PBS), and re-suspended in 0.5 mL of PBS.

Cell acquisition was performed using a BD FACSCanto II cytometer (Becton Dickinson, Franklin Lakes, NJ, USA), equipped with three lasers: 488 nm blue, 633 nm red, and 405 nm violet. Analyses were conducted using BD FACSDiva Software (Becton Dickinson). We acquired at least 100,000 events for each sample-MoAb combination. To detect dead cells and test the erythrocyte lysis efficiency, we performed additional staining with SYTO16-FITC (Molecular Probes, Leiden, The Netherlands), a live-cell-permeant nucleated-cell dye, and 7AAD-PC5.5 dye (Beckman Coulter, Inc., Brea, CA, USA). MDD was then expressed as the percentage of cells characterized by a LBL immunophenotype among BM/PB nucleated cells.

### 2.3. NOTCH1/FBXW7 Mutational Analysis

Genomic DNA was obtained from tumor tissues using the QIAmp DNA Mini kit (Qiagen, Hilden, Germany), according to the manufacturer’s instructions.

Mutational analyses of candidate genes were performed on *NOTCH1* exons 26 and 27, encoding for the heterodimerization domain, *NOTCH1* exon 34, encoding for the transactivation and PEST domains, and *FBXW7* exons 9, 10 and 12. Mutation hot spots were amplified as previously reported [10]. PCR amplicons were purified using the IllustraExoProStar^TM^ 1-Step reagent (Cytiva, Marlborough, MA, USA) and sequenced on a 3500 DX Genetic Analyzer (ThermoFisher Scientific, Waltham, MA, USA).

### 2.4. Statistical Analysis

Survival analyses were performed according to the Kaplan–Meier (KM) method and the survival functions obtained were compared using the log-rank test [15]. Overall survival (OS) was calculated from the date of diagnosis to the date of death, whatever the cause. Progression-free survival (PFS) was calculated from the date of diagnosis to the date of the first event (relapse, refractory disease, disease progression or death from any cause) or to the date of the last follow-up. Univariate comparisons were performed according to the log-rank test (Supplementary Tables S1 and S2). Median follow-up was calculated by the reverse KM method. All *p*-values are two-sided, with a type I error rate fixed at 0.05.

Data analyses were performed by using SAS statistical analysis software (SAS-PC, version 9.4, SAS institute, Cary, NC, USA).

## 3. Results

### 3.1. Clinical Features

The study cohort of 132 pediatric LBL patients included 40 females and 92 males. The median age at diagnosis was 8.3 years (range 1.0–18.0 years). Of them, 127 patients (97%) achieved complete remission (CR), while five patients (3%) showed a refractory disease. Two out of five therapy-resistant patients died from disease progression, whereas the other three were currently alive at follow-up. Among the 127 patients in remission, 16/127 (13%) relapsed at a median time of one year (range 0.1–5.5 years) from diagnosis. Eleven out of the 16 relapsed patients died from disease progression (one patient after undergoing hematopoietic stem cell transplantation), while the other five are currently alive in CR.

Overall, at the date of the last follow-up, 119/132 were alive in CR, 13/132 patients died of progressive disease (11/13 relapsed and 2/13 had a refractory disease), while one patient developed secondary malignancy (thyroid cancer) four years after diagnosis. No therapy-related toxic deaths were observed. The overall PFS (± SE) at three years was 83% (± 4%) with a median follow-up of 4.3 years (range 0.2–9 years).

Three patients out of 132 were in stage I at diagnosis, and six patients were in stage II. No events were recorded in patients in stages I and II, and the four-year PFS was 100%. Among the 74 patients in stage III at diagnosis, 18 events occurred, and the four-year PFS (± SE) was 75% (± 5%). In the 49 patients classified in stage IV, three events occurred with a four-year PFS (± SE) of 93% (± 4%).

No statistically significant difference in PFS was observed among LBL patients considering the following parameters at diagnosis: sex, median age, immunophenotype, mediastinal involvement, stage, morphological infiltration of BM, CNS involvement and treatment protocol (Supplementary Tables S1 and S2).

The characteristics of the study population are reported in Table 1.

#### 3.1.1. MDD Analysis in LBL Patients

Multiparametric flow cytometric (MFC) analysis of MDD was performed on unilateral or bilateral BM and/or PB samples in all 132 patients. In 72/132 patients (55%), a positive MFC-MDD was detected (range 0.02–70.00%, median value 3.65% and average value 7.16%): of these, one patient was in stage I, one in stage II, 32/72 in stage III and 38/72 in stage IV (7/38 with CNS involvement); regarding the immunophenotype, 64/72 were T-LBL while 8/72 were pB-LBL. Ten out of 72 patients (15%) experienced an unfavorable event: 8/10 relapsed, 1/10 showed refractory disease and 1/10 developed a secondary malignancy. As for the 60/132 MDD negative patients, 43 were T-LBL and 17 were pB-LBL.

#### 3.1.2. *NOTCH1/FBXW7* Mutational Analysis in T-LBL Patients

The genetic analysis of N/F genes was performed on 58/107 T-LBL patients for which the tumor tissue was available. The N/F genes detected mutations in 24/58 patients (41%, N/F^mut^). Of these, 20/24 presented a genetic alteration exclusively on *NOTCH1*, 1/24 exclusively on *FBXW7* and 3/24 on both the genes (Supplementary Table S3, Supplementary Figures S1 and S2); 18/24 patients were in stage III at diagnosis and 6 were in stage IV (1/6 with CNS involvement). It is worth noting that none of N/F^mut^ patients experienced an unfavorable event, whereas 10 events occurred among the 34 nonmutated (N/F^wt^) patients (7/10 patients relapsed and 3/10 showed refractory disease).

### 3.2. Stratification of Patients Based on MDD at Diagnosis

The prognostic significance of MDD at diagnosis was assessed by using the 3% of LBL cells as cut-off level, as previously published by our group [8].

**Figure 1:**
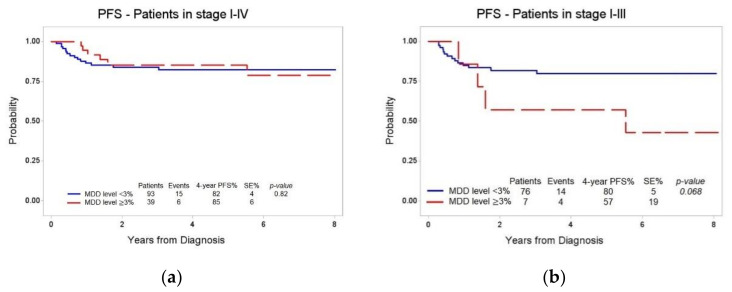
Four-year PFS according to MDD levels above or below the 3% cut-off: (**a**) all LBL patients with available MDD results; (**b**) LBL patients in stage I–III.

In 93/132 patients, MDD was <3%: 15/93 patients experienced an event, and the four-year PFS (± SE) was 82% (± 4%). In 39 patients with MDD ≥ 3%, there were six events with a four-year PFS (± SE) of 85% (± 6%). No statistically significant difference in PFS was observed (Figure 1a, *p* = 0.82). Considering the subgroup of stage IV patients, including those with CNS involvement, MDD was <3% in 17/49, and the four-year PFS (± SE) was 94% (± 6%). Among the 32/49 patients with MDD ≥3%, two experienced an event, and the four-year PFS (± SE) was 93% (± 5%), (*p* = 0.98) (Supplementary Figure S3).

To better evaluate the prognostic value of MDD, we decided to focus on patients without morphologically highlighted BM involvement at diagnosis (stages I, II and III), which were also uniformly treated, using the 3% of LBL cells as cut-off for patients’ stratification. In 76/83 patients, MDD level was <3%: 14/76 patients experienced an event, and the four-year PFS (± SE) was 80% (± 5%). Conversely, among the seven patients with MDD ≥ 3%, 4/7 experienced an event, with a four-year PFS (± SE) of 57% (± 19%). Even though the difference in PFS did not reach statistical significance (Figure 1b, *p* = 0.068), it is worth noting that more than half of the patients with MDD ≥ 3% had a poorer prognosis.

### 3.3. Stratification of Patients Based on NOTCH1/FBXW7 Mutational Status

**Figure 2:**
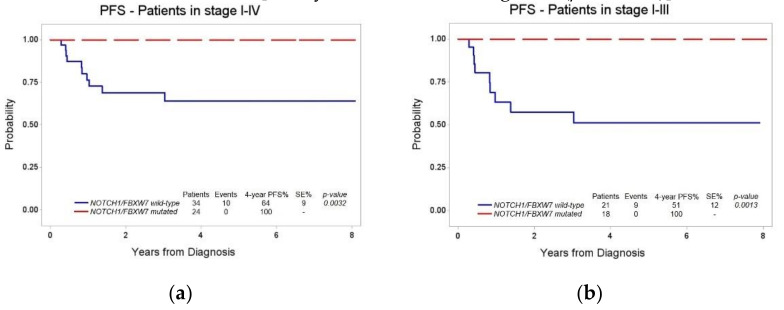
Four-year PFS according to *NOTCH1/FBXW7* mutational status: (**a**) all T-LBL patients with available N/F mutations data; (**b**) T-LBL patients in stage I–III.

To evaluate the prognostic role of *NOTCH1/FBXW7* mutational status, we conducted a first survival analysis taking into account all the patients for which the genetic data were available (*n* = 58). Of them, 34/58 were N/F^wt^: 10/34 patients experienced an event, and the four-year PFS (± SE) was 64% (± 9%). Conversely, none of the 24 N/F^mut^ patients experienced an event (four-year PFS 100%), thus confirming that the presence of N/F mutation in LBL tumor tissue translates into a significantly more favorable outcome (Figure 2a, *p* = 0.0032).

We next focused on uniformly treated T-LBL patients in stage I, II and III with available N/F and MDD results (*n* = 39): 21/39 were N/F^wt^ and 9/21 patients experienced an event, with a four-year PFS (± SE) of 51% (± 12%). Among the 18/39 N/F^mut^ patients, no event was recorded (four-year PFS 100%). The difference in PFS between N/F^wt^ and N/F^mut^ patients in stages I–III remained statistically significant (Figure 2b, *p* = 0.0013).

### 3.4. Combined Stratification of Patients Based on MDD at Diagnosis and NOTCH1/FBXW7 Mutational Status

We thus performed a survival analysis in T-LBL patients in stage I–III, matching the results of the 3% cut-off MDD analysis with the results of the *NOTCH1/FBXW7* genetic analysis.

**Figure 3:**
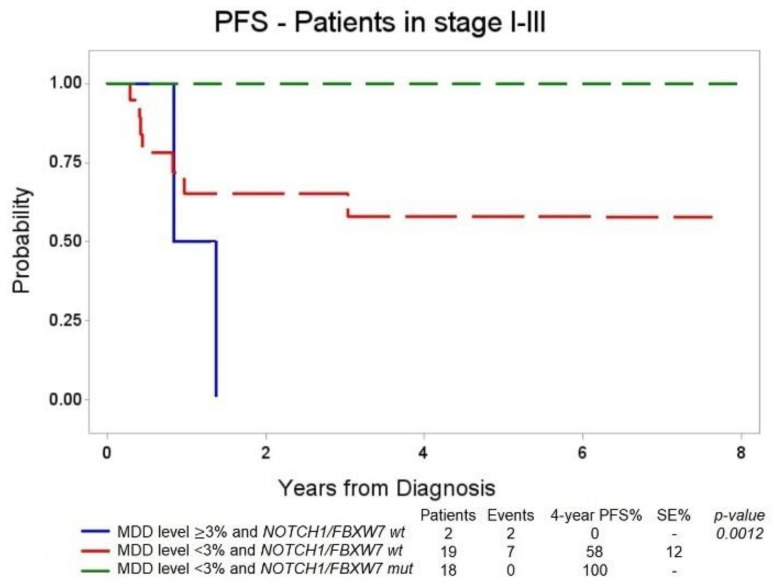
Four-year PFS according to MDD levels and *NOTCH1/FBXW7* mutational status in uniformly treated T-LBL patients without morphological BM involvement (stages I–III).

Among MDD positive ≥3% patients, none of them was N/F^mut^, and the only two patients with MDD ≥ 3% and N/F^wt^ both experienced an event. In 37/39 patients with MDD < 3%, 19/37 patients were N/F^wt^ and 7/19 experienced an event [four-year PFS 58% (± 12%)], whereas none of the 18 patients with MDD < 3% and 18 N/F^mut^ relapsed or exhibited treatment resistance [four-year PFS of 100%, Figure 3 (*p* = 0.0012)].

Stratification of patients based on MDD at diagnosis and N/F mutational status performed according to OS overlapped results of PFS analysis (Supplementary Figures S4–S6).

## 4. Discussion

In the present study, we confirmed the high frequency of disease dissemination at diagnosis in a large cohort of LBL pediatric patients treated in AIEOP centers. The prognostic potential of MDD was assessed by stratifying patients on the basis of the 3% cut-off level, according to previously published data [8]. When all the patients with MDD results were considered, no statistically significant difference was observed in the four-year PFS of patients with MDD values above or below the chosen cut-off. A possible interpretation of this finding can be sought by studying the clinical characteristics of the study cohort. Observing how the events are distributed in relation to the disease stage, it is noteworthy that most of the events (18/21) occurred in stage III patients. This can be explained by the fact that patients in stage IV with CNS involvement treated according to both the AIEOP LNH-97 and the international EURO-LB02 protocols underwent a treatment intensification involving additional intrathecal therapies associated or not with cranial radiotherapy, aimed to provide a prophylaxis for local recurrence. To eliminate this treatment bias, we conducted a second stratification taking into account only stage I–III patients, and, in line with previously published data [8], we observed a clear reduction in the four-year PFS of patients with MDD ≥ 3% compared to patients with MDD < 3%. However, the number of patients with available MDD results, particularly for the group with MDD above the cut-off value, is still reduced. In light of this, we believe that further studies are needed to confirm the prognostic potential of MDD in LBL. When data from larger patients’ populations will be available, different cut-offs to stratify patients could be also considered to find the best way to introduce this parameter in clinical practice.

We also evaluated the prognostic role of the mutational status of *NOTCH1/FBXW7* genes, both including all the patients with available tumor tissue for the genetic analyses and only stage I–III patients. In both the cases, the presence of N/F mutation was associated with a significantly better clinical outcome. Indeed, none of the patients with mutations in N/F hot-spot exons experienced an event.

Our results confirm the strong prognostic power of the mutational status of NOTCH1 pathway, which is hypothesized to be influenced by the therapeutic regimen adopted. These results confirmed previous data used for the construction of a patients’ stratification model in the new treatment protocol for pediatric LBL (clinical trial NCT04043494).

Interestingly, here for the first time we analyzed the combined prognostic power of both MDD and N/F mutational status for pediatric LBL patients’ stratification, and we were able to classify patients into different risk groups with significantly different prognosis—low risk: MDD < 3% and N/F^mut^ (four-year PFS 100%); intermediate risk: MDD < 3% and N/F^wt^ (four-year PFS 58% ± 12); high risk: MDD ≥ 3% and N/F^wt^ (four-year PFS 0%).

One limitation of our study is that we had the opportunity to analyze both MDD and N/F mutational status only in subset of T-LBL patients with available BM/PB, due to the lack of tumor tissue samples. This is primarily due to the localization of LBL disease, which is characterized by mediastinal masses not safely accessible to obtain material for biological investigations. However, because N/F mutational analysis will be mandatory for T-LBL patients’ stratification in the forthcoming international trial for pediatric LBL treatment, the analysis of a larger cohort of patients will be soon feasible, allowing us to better define the predictive power of the combination of the two factors.

In conclusion, the results described in this work demonstrate that, unlike other forms of pediatric lymphoma, such as Burkitt lymphoma [16] and anaplastic large cell lymphoma [17] for which MDD represents an independent prognostic factor, MDD seems to play a less relevant prognostic role in T-LBL when compared to the mutational status of *NOTCH1/FBXW7*. Although the modalities in which N/F mutations influence response to treatment are not yet fully known, it is believed that the hyperactivation of NOTCH1 alters the chemosensitivity of neoplastic cells—making them more sensitive to therapy—and that the effect of this modulation may depend on the polichemotherapy regimen in terms of type of drugs, posology and administration protocol.

In any case, our preliminary results suggest that the combination of MDD and N/F mutational data could identify patients’ subgroups with significantly different prognosis, even though in our study cohort, some groups are represented by very few patients. In the future, it will be important to prospectively analyze MDD and N/F mutational status in all the patients enrolled in the LBL 2018 treatment protocol in order to be able to validate these observations in an independent cohort of patients and to enable a more and more personalized approach to LBL treatment. Moreover, the evaluation of minimal residual disease during treatment may also play a prognostic role and could contribute in further treatment optimization strategies.

## Figures and Tables

**Table 1:** Clinical characteristics and outcome of patients.

	Patients (*n* = 132)	%
**Gender**		
Male	92	69.7
Female	40	30.3
**Median age at diagnosis**		
≤ 8.3 years	66	50
> 8.3 years	66	50
**Immunophenotype**		
T	107	81.1
pB	25	18.9
**Mediastinal involvement**		
Yes	89	67.4
No	43	32.6
**BM involvement**		
Yes	43	32.6
No	89	67.4
**Stage**		
I	3	2.3
II	6	4.5
III	74	56.1
IV CNS-	37	28
IV CNS+	12	9.1
**MFC-MDD**		
Positive	72	54.5
Negative	60	45.5
Mutational status *		
N/F^mut^	24	41.4
N/F^wt^	34	58.6
**Events**		
Relapse	16	12.1
Refractory disease	5	3.8
Secondary malignancy	1	0.8
**Death**		
No. Patients	13	9.8

mut, mutated; wt, wild type; CNS, central nervous system; MFC-MDD, multiparametric flow cytometry minimal disseminated disease; * performed only on a subgroup of 58 patients.

## Supplementary Materials

The following are available online at https://www.mdpi.com/article/10.3390/diagnostics11091594/s1. Supplementary Table S1: Univariate analysis on the whole cohort of 132 LBL patients. Supplementary Table S2: Univariate analysis on the 83 LBL patients in stage I−III. Supplementary Table S3: List of NOTCH1/FBXW7 mutations identified in 24 T-LBL patients. Supplementary Figure S1: Multiple sequence alignment of NOTCH1 wild-type and variants identified in the present study. Supplementary Figure S2: Multiple sequence alignment of FBXW7 wild-type and variants identified in the present study. Supplementary Figure S3: 4-years PFS of stage IV LBL patients (N = 49) stratified according to MDD levels above or below the 3% cut-off. Supplementary Figure S4: 4-years OS according to MDD levels above or below the 3% cut-off: (a) all LBL patients with available MDD results; (b) LBL patients in stage I−III. Supplementary Figure S5: 4-years OS according to *NOTCH1/FBXW7* mutational status: (a) all T-LBL patients with available N/F mutations data; (b) T-LBL patients in stage I−III. Supplementary Figure S6: 4-years OS according to MDD levels and *NOTCH1/FBXW7* mutational status in uniformly treated T-LBL patients without morphological BM involvement (stages I−III).

## References

[1] Burkhardt B., Hermiston M.L. (2019). Lymphoblastic lymphoma in children and adolescents: Review of current challenges and future opportunities. Br. J. Haematol..

[2] Burkhardt B., Zimmermann M., Oschlies I., Niggli F., Mann G., Parwaresch R., Riehm H., Schrappe M., Reiter A. (2005). The impact of age and gender on biology, clinical features and treatment outcome of non-Hodgkin lymphoma in childhood and adolescence. Br. J. Haematol..

[3] Oschlies I., Burkhardt B., Chassagne-Clement C., D’Amore E.S., Hansson U., Hebeda K., Mc Carthy K., Kodet R., Maldyk J., Müllauer L. (2011). Diagnosis and immunophenotype of 188 pediatric lymphoblastic lymphomas treated within a randomized prospective trial: Experiences and preliminary recommendations from the european childhood lymphoma pathology panel. Am. J. Surg. Pathol..

[4] Termuhlen A.M., Smith L.M., Perkins S.L., Lones M., Finlay J.L., Weinstein H., Gross T.G., Abromowitch M. (2013). Disseminated lymphoblastic lymphoma in children and adolescents: Results of the COG A5971 trial: A report from the Children’s Oncology Group. Br. J. Haematol..

[5] Landmann E., Burkhardt B., Zimmermann M., Meyer U., Woessmann W., Klapper W., Wrobel G., Rosolen A., Pillon M., Escherich G. (2017). Results and conclusions of the European intergroup EURO-LB02 trial in children and adolescents with lymphoblastic lymphoma. Haematologica.

[6] Schmidt E., Burkhardt B. (2013). Lymphoblastic lymphoma in childhood and adolescence. Pediatr. Hematol. Oncol..

[7] Coustan-Smith E., Sandlund J.T., Perkins S.L., Chen H., Chang M., Abromowitch M., Campana D. (2009). Minimal disseminated disease in childhood T-cell lymphoblastic lymphoma: A report from the Children’s Oncology Group. J. Clin. Oncol..

[8] Mussolin L., Buldini B., Lovisa F., Carraro E., Disarò S., Nigro L.L., d’Amore E.S.G., Pillon M., Basso G. (2015). Detection and role of minimal disseminated disease in children with lymphoblastic lymphoma: The AIEOP experience. Pediatr. Blood Cancer.

[9] Callens C., Baleydier F., Lengline E., Ben Abdelali R., Petit A., Villarese P., Cieslak A., Minard-Colin V., Rullier A., Moreau A. (2012). Clinical impact of NOTCH1 and/or FBXW7 mutations, FLASH deletion, and TCR status in pediatric T-cell lymphoblastic lymphoma. J. Clin. Oncol..

[10] Bonn B.R., Rohde M., Zimmermann M., Krieger D., Oschlies I., Niggli F., Wrobel G., Attarbaschi A., Escherich G., Klapper W. (2013). Incidence and prognostic relevance of genetic variations in T-cell lymphoblastic lymphoma in childhood and adolescence. Blood.

[11] Pillon M., Aricò M., Mussolin L., Carraro E., Conter V., Sala A., Buffardi S., Garaventa A., D’Angelo P., Lo Nigro L. (2015). Long-term results of the AIEOP LNH-97 protocol for childhood lymphoblastic lymphoma. Pediatr. Blood Cancer.

[12] Swerdlow S.H., Campo E., Pileri S.A., Lee Harris N., Stein H., Siebert R., Advani R., Ghielmini M., Salles G.A., Zelenetz A.D. (2016). The 2016 revision of the World Health Organization classification of lymphoid neoplasms. Blood.

[13] Basso G., Buldini B., De Zen L., Orfao A. (2001). New methodologic approaches for immunophenotyping acute leukemias. Haematologica.

[14] Dworzak M.N., Gaipa G., Ratel R., Veltroni M., Schumich A., Maglia O., Karawajew L., Benetello A., Pötschger U., Husak Z. (2008). Standardization of flow cytometric minimal residual disease evaluation in acute lymphoblastic leukemia: Multicentric assessment is feasible. Cytom. Part B Clin. Cytom..

[15] Bewick V., Cheek L., Ball J. (2004). Statistics review 12: Survival analysis. Crit. Care.

[16] Mussolin L., Pillon M., D’Amore E.S.G., Conter V., Piglione M., Lo Nigro L., Garaventa A., Buffardi S., Aricò M., Rosolen A. (2011). Minimal disseminated disease in high-risk Burkitt’s lymphoma identifies patients with different prognosis. J. Clin. Oncol..

[17] Mussolin L., Le Deley M.C., Carraro E., Damm-Welk C., Attarbaschi A., Williams D., Burke A., Horibe K., Nakazawa A., Wrobel G. (2020). Prognostic factors in childhood anaplastic large cell lymphoma: Long term results of the international alcl99 trial. Cancers.

