# Supplementary material for: Prognostic Role of Minimal Disseminated Disease and NOTCH1/FBXW7 Mutational Status in Children with Lymphoblastic Lymphoma: The AIEOP Experience
Source: Diagnostics (Basel). 2021 Sep 1;11(9):1594. doi: 10.3390/diagnostics11091594 (PMC8468850; doi:10.3390/diagnostics11091594)

Supplementary Table S1. Univariate analysis on the whole cohort of 132 LBL patients.

Characteristics	Categories	N° patients	Events	4-year PFS % (SE%)	Univariate p-value
Treatment protocol	LNH97	29	6	82 (7)	0.72
	EuroLB02	103	15	84 (4)	
Age	<8.3 years	66	11	83 (5)	0.86
	≥8.3 years	66	10	83 (5)	
Gender	Male	92	16	81 (4)	0.46
	Female	40	5	87 (5)	
Stage	I+II	9	0	100 -	0.18
	III+IV	123	21	79 (4)	
Immunophenotype	T	107	17	83 (4)	0.87
	pB	25	4	82 (8)	
BM involvement	Yes	43	2	92 (4)	0.0686
	No	89	19	79 (5)	
Mediastinal involvement	Yes	89	15	82 (4)	0.61
	No	43	6	85 (6)	
CNS involvement	Yes	12	2	83 (11)	0.77
	No	120	19	83 (4)	
MDD	<3% cut-off	93	15	82 (4)	0.82
	≥3% cut-off	39	6	85 (6)	
Mutational status*	N/Fmut	24	0	100	0.0032
	N/Fwt	34	10	64	

T: T-cell; pB: B-cell precursor; MDD: minimal disseminated disease; N/Fmut: *NOTCH/FBXW7* mutated; N/Fwt: *NOTCH/FBXW7* wild-type.
*data were available only for 58 T-LBL patients.

Supplementary Table S2. Univariate analysis on the 83 LBL patients in stage I-III.

Characteristics	Categories	N° patients	Events	4-year PFS % (SE%)	Univariate p-value
Treatment protocol	LNH97	19	5	78 (10)	0.84
	EuroLB02	64	13	77 (5)	
Age	< 8.3 years	43	9	79 (7)	0.87
	≥ 8.3 years	40	9	79 (7)	
Gender	Male	61	13	78 (6)	0.46
	Female	22	5	77 (9)	
Immunophenotype	T	65	14	78 (5)	0.94
	pB	18	4	74 (12)	
MDD	<3% cutoff	76	14	80 (5)	0.068
	≥3% cutoff	7	4	57 (19)	
Mediastinal involvement	Yes	58	13	76 (6)	0.69
	No	25	5	80 (9)	
Mutational status*	N/Fmut	18	0	100	0.0013
	N/Fwt	21	9	64	

T: T-cell; pB: B-cell precursor; MDD: minimal disseminated disease; N/Fmut: *NOTCH/FBXW7* mutated; N/Fwt: *NOTCH/FBXW7* wild-type.
*data were available only for 39 T-LBL patients.

Supplementary Table S3: List of NOTCH1/FBXW7 mutations identified in 24 T-LBL patients. Sequence numbering is based on GenBank accessions NM_017617.4/NP_060087.3 for NOTCH1 and NM_033632.3/NP_361014.1 for FBXW7.

	***NOTCH1* HD domain**		***NOTCH1* PEST domain**		***FBXW7***	
**patient**	**DNA**	**protein**	**DNA**	**protein**	**DNA**	**protein**
TLBL6	c.4721T>C	p.L1574P			c.1393C>T<br>c.2065C>T	p.R465C<br>p.R689W
TLBL21			c.7541-42delCT	p.S2513fs*3		
TLBL22	c.4733T>A	p.V1578E	c.7354-55insGA	p.L2451fs*25		
TLBL26	c.4732-34del	p.V1578del	c.7538-39del	p.P2514*		
TLBL30			c.7397C>T	p.T2466M		
TLBL36						
TLBL39	c.4754T>C	p.L1585P				
TLBL50	c.4775T>C	p.F1592S				
TLBL58					c.1552G>A	p.A518T
TLBL61	c.4793G>C	p.R1598P				
TLBL62	c.4721T>C	p.L1574P				
TLBL64	c.4799T>C	p.L1600P				
TLBL68	c.4721T>C	p.L1574P	c.7130-37del>insTCGCGAA	p.P2410fs*10		
TLBL72			c.7389-401del>insTCC	p.A2463fs*9	c.1394G>A	p.R465H
TLBL78	c.5101G>C<br>c.5153T>C	p.A1701P<br>p.I1718T	c.7541-42del	p.S2513fs*3		
TLBL88	c.4799T>C	p.L1600P				
TLBL90	c.4775-76insTGGCCC	p.F1592insGP			c.1429G>A	p.G477S
TLBL94	c.4793G>C	p.R1598P				
TLBL103	c.4793G>C	p.R1598P				
TLBL106	c.4732-34del	p.V1578del				
TLBL107	c.4775-76insGATACC	p.F1592>LIT				
TLBL118	c.4810-12del	p.V1604del	c.7533-7541del	p.P2512*		
TLBL119			c.7327G>AGCCCC	p.D2442fs*35		
TLBL125			c.7400 C>A	p.S2467*		

Supplementary Figure S1: Multiple sequence alignment of NOTCH1 wild-type and variants identified in the present study. Number of first and last depicted amino acids of reference sequence (ref_seq) are indicated based on NP_060087.3. Sequence differences caused by missense mutations, insertions or frame-shifts are shown.
* stop codon
# deleted codon

NOTCH1 HD domain

```
            1540                                                                                            1636
ref_seq     FSDGHCDQGCNSAECEWDGLDCAEHVPERLAAGTLVVVVLMPPEQLRNSSFHF---LRELSRVLHTNVVFKRDAHGQQMIFPYYGREEELRKHPIKRAAE
TLBL6       ----------------------------------P------------------   --------------------------------------------
TLBL62      ----------------------------------P------------------   --------------------------------------------
TLBL68      ----------------------------------P------------------   --------------------------------------------
TLBL26      --------------------------------------#--------------   --------------------------------------------
TLBL106     --------------------------------------#--------------   --------------------------------------------
TLBL22      --------------------------------------E--------------   --------------------------------------------
TLBL39      ---------------------------------------------P-------   --------------------------------------------
TLBL50      ----------------------------------------------------S   --------------------------------------------
TLBL107     -----------------------------------------------------ITL--------------------------------------------
TLBL90      -----------------------------------------------------G-P--------------------------------------------
TLBL61      -----------------------------------------------------   -----P--------------------------------------
TLBL94      -----------------------------------------------------   -----P--------------------------------------
TLBL103     -----------------------------------------------------   -----P--------------------------------------
TLBL64      -----------------------------------------------------   -------P------------------------------------
TLBL88      -----------------------------------------------------   -------P------------------------------------
TLBL118     -----------------------------------------------------   -----------#--------------------------------
TLBL78      -----------------------------------------------------   --------------------------------------------

            1632                                                                                            1731
ref_seq     KRAAEGWAAPDALLGQVKASLLPGGSEGGRRRRELDPMDVRGSIVYLEIDNRQCVQASSQCFQSATDVAAFLGALASLGSLNIPYKIEAVQSETVEPPPP
TLBL6       ----------------------------------------------------------------------------------------------------
TLBL62      ----------------------------------------------------------------------------------------------------
TLBL68      ----------------------------------------------------------------------------------------------------
TLBL26      ----------------------------------------------------------------------------------------------------
TLBL106     ----------------------------------------------------------------------------------------------------
TLBL22      ----------------------------------------------------------------------------------------------------
TLBL39      ----------------------------------------------------------------------------------------------------
TLBL50      ----------------------------------------------------------------------------------------------------
TLBL107     ----------------------------------------------------------------------------------------------------
TLBL90      ----------------------------------------------------------------------------------------------------
TLBL61      ----------------------------------------------------------------------------------------------------
```

```
TLBL94      ----------------------------------------------------------------------------------------------------
TLBL103     ----------------------------------------------------------------------------------------------------
TLBL64      ----------------------------------------------------------------------------------------------------
TLBL88      ----------------------------------------------------------------------------------------------------
TLBL118     ----------------------------------------------------------------------------------------------------
TLBL78      ---------------------------------------------------------------------P----------------T-------------
```

NOTCH1 PEST DOMAIN

```
             2379                                                                                            2478
ref_seq      LVQTQQVQPQNLQMQQQNLQPANIQQQQSLQPPPPPPQPHLGVSSAASGHLGRSFLSGEPSQADVQPLGPSSLAVHTILPQESPALPTSLPSSLVPPVTA
TLBL30       ---------------------------------------------------------------------------------------M------------
TLBL21       ----------------------------------------------------------------------------------------------------
TLBL78       ----------------------------------------------------------------------------------------------------
TLBL26       ----------------------------------------------------------------------------------------------------
TLBL118      ----------------------------------------------------------------------------------------------------
TLBL119      ----------------------------------------------------------------SPCSHWAPAAWWCTLFCPRRAPPCPRRCHPRWSHP*
TLBL22       -------------------------------------------------------------------------GRCTLFCPRRAPPCPRRCHPRWSHP*
TLBL72       -------------------------------------------------------------------------------------PCHPRWSHP*
TLBL125      -----------------------------------------------------------------------------------------*
TLBL68       --------------------------------REHHHSRTLA*
```

```
             2479                                                                        2556
ref_seq      AQFLTPPSQHSYSSPVDNTPSHQLQVPEHPFLTPSPESPDQWSSSSPHSNVSDWSEGVSSPPTSMQSQIARIPEAFK*
TLBL30       ------------------------------------------------------------------------------
TLBL21       -----------------------------------RVP*
TLBL78       -----------------------------------RVP*
TLBL26       -----------------------------------*
TLBL118      ----------------------------------*
LBL119
TLBL22
TLBL72
TLBL125
TLBL68
```

Supplementary Figure S2: Multiple sequence alignment of FWBX7 wild-type and variants identified in the present study. Number of first and last depicted amino acids of reference sequence (ref_seq) are indicated based on NP_361014.1. Sequence differences caused by missense mutations are shown.
* stop codon

```
             422                                                                                              521
ref_seq      GGVWSSQMRDNIIISGSTDRTLKVWNAETGECIHTLYGHTSTVRCMHLHEKRVVSGSRDATLRVWDIETGQCLHVLMGHVAAVRCVQYDGRRVVSGAYDF
TLBL6        -------------------------------------------C--------------------------------------------------------
TLBL72       -------------------------------------------H--------------------------------------------------------
TLBL90       -------------------------------------------------------S--------------------------------------------
TLBL58       ------------------------------------------------------------------------------------------------T---


             618                                                                                     708
ref_seq      QGPNKHQSAVTCLQFNKNFVITSSDDGTVKLWDLKTGEFIRNLVTLESGGSGGVVWRIRASNTKLVCAVGSRNGTEETKLLVLDFDVDMK*
TLBL6        -----------------------------------------------------------------------W-------------------
TLBL72       -------------------------------------------------------------------------------------------
TLBL90       -------------------------------------------------------------------------------------------
TLBL58       -------------------------------------------------------------------------------------------
```

Supplementary Figure S3. 4-year PFS of stage IV LBL patients (N=49) stratified according to MDD levels above or below the 3% cut-off

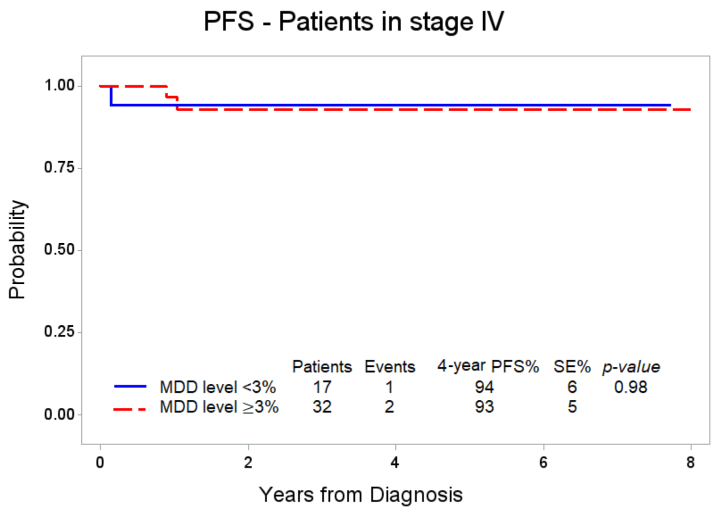

The same analysis on stage IV patients without CNS involvement (N=37) showed a 4-year PFS of 100% and 96% (± 4%) for patients with MDD <3% and ≥3% (p-value 0.56), respectively.
As for stage IV patients with CNS involvement (N=12), 4-year PFS was 88% (± 13%) for patients with MDD <3% (8/12), whereas patients with MDD≥3% (4/12) had a 4-year PFS of 75% (± 25%). No statistically significant difference was observed (p-value 0.69).

Supplementary Figure S4. 4-year OS according to MDD levels above or below the 3% cut-off: (a) all LBL patients with available MDD results; (b) LBL patients in stage I-III.

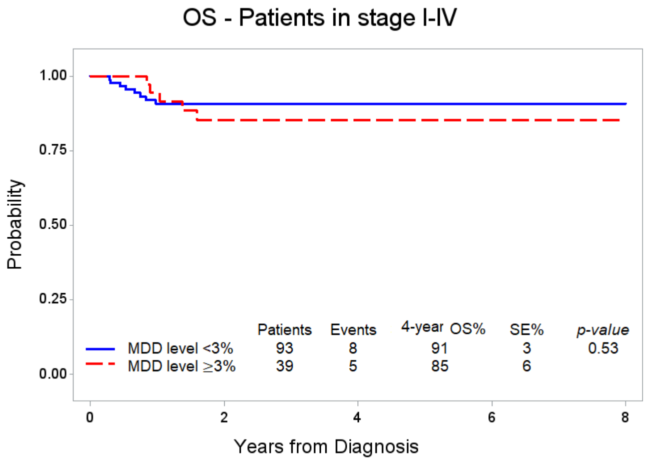

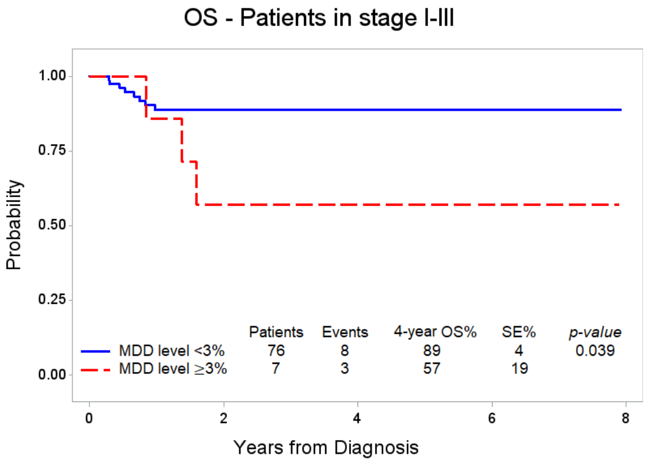

(a) (b)

Supplementary Figure S5. 4-year OS according to *NOTCH1/FBXW7* mutational status: (a) all T-LBL patients with available N/F mutations data; (b) T-LBL patients in stage I-III.

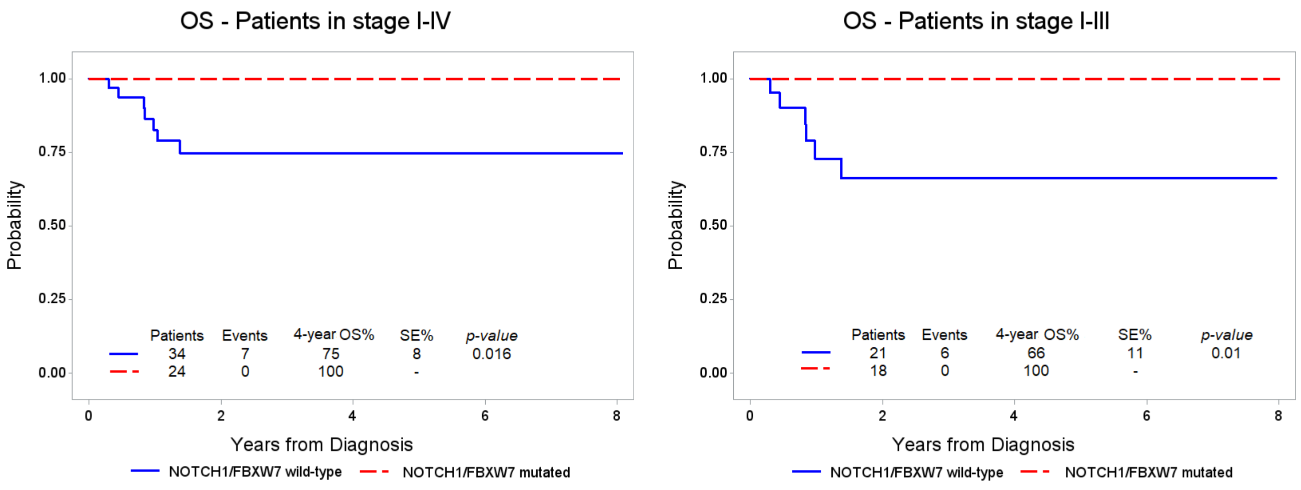

(a) (b)

Supplementary Figure S6. 4-year OS according to MDD levels and *NOTCH1/FBXW7* mutational status in uniformly treated T-LBL patients without morphological BM involvement (stages I-III).

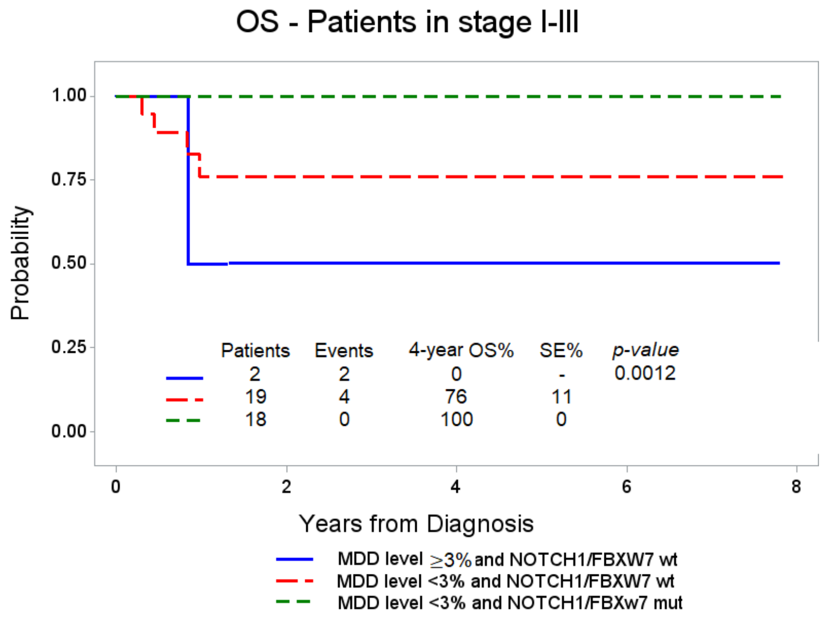

Supplement: Supplementary file 1 [file diagnostics-11-01594-s001.zip › diagnostics-1349299-supplementary.pdf]
